# Climate, Cattle Rearing Systems and African Animal Trypanosomosis Risk in Burkina Faso

**DOI:** 10.1371/journal.pone.0049762

**Published:** 2012-11-14

**Authors:** Soumaïla Pagabeleguem, Mamadou Sangaré, Zakaria Bengaly, Massouroudin Akoudjin, Adrien M. G. Belem, Jérémy Bouyer

**Affiliations:** 1 Centre International de Recherche-Développement sur l'Elevage en Zone subhumide (CIRDES), Bobo-Dioulasso, Burkina Faso; 2 Université Polytechnique de Bobo-Dioulasso (UPB), Bobo-Dioulasso, Burkina Faso; 3 Institut Sénégalais de Recherches Agricoles, Laboratoire National d'Elevage et de Recherches Vétérinaires, Hann, Dakar, Sénégal; 4 UMR Contrôle des Maladies Animales Exotiques et Emergentes, Centre de Coopération Internationale en Recherche Agronomique pour le Développement (CIRAD), Campus International de Baillarguet, Montpellier, France; Obihiro University of Agriculture and Veterinary Medicine, United States of America

## Abstract

**Background:**

In sub-Saharan countries infested by tsetse flies, African Animal Trypanosomosis (AAT) is considered as the main pathological constraint to cattle breeding. Africa has known a strong climatic change and its population was multiplied by four during the last half-century. The aim of this study was to characterize the impact of production practices and climate on tsetse occurrence and abundance, and the associated prevalence of AAT in Burkina Faso.

**Methodology/Principal Findings:**

Four sites were selected along a South-north transect of increasing aridity. The study combines parasitological and entomological surveys. For the parasitological aspect, blood samples were collected from 1,041 cattle selected through a stratified sampling procedure including location and livestock management system (long transhumance, short transhumance, sedentary). Parasitological and serological prevalence specific to livestock management systems show a gradual increase from the Sahelian to the Sudano-Guinean area (P<0.05). Livestock management system had also a significant impact on parasitological prevalence (P<0.05). Tsetse diversity, apparent densities and their infection rates overall decreased with aridity, from four species, an apparent density of 53.1 flies/trap/day and an infection rate of 13.7% to an absence at the northern edge of the transect, where the density and diversity of other biting flies were on the contrary highest (p<0.001).

**Conclusions/Significance:**

The climatic pressure clearly had a negative impact on tsetse abundance and AAT risk. However, the persistency of tsetse habitats along the Mouhoun river loop maintains a high risk of cyclical transmission of *T. vivax*. Moreover, an “epidemic mechanical livestock trypanosomosis” cycle is likely to occur in the northern site, where trypanosomes are brought in by cattle transhuming from the tsetse infested area and are locally transmitted by mechanical vectors. In Burkina Faso, the impact of tsetse thus extends to a buffer area around their distribution belt, corresponding to the herd transhumance radius.

## Introduction

The climatic change and its impact on environments vary extensively in space and time [1], due to local socio-cultural and biophysical conditions. However, the increase of the frequency and amplitude of extreme climatic events (extreme temperatures, droughts, flooding, etc.) is generally fast, like the extreme vulnerability of West African small producers to cope with these changes. These events are not caused by natural variations of the climate only: they are exemplified by an increased exploitation of natural resources, particularly land use [2]. For example, from some 30 million inhabitants in 1900, West African population increased to 306 million in 2010, and could reach between 550 and 700 million in 2050 [3]. The development of extensive agriculture and the increase in human density are associated to landscape fragmentation in the Mouhoun river basin [4]. Associated to extreme climatic events, they have huge consequences on the environment and sustainable development, particularly for African producers who depend strongly on ecosystem services like natural grazing [5]. These changes have also an impact on vectors' environment, and thus on their geographical distribution and density. This situation causes a modification of the hosts-vector contacts and hence, of the epidemiology of vector diseases, particularly African Animal Trypanosomoses (AAT) [6].

Indeed, these diseases represent the main constraint to the development of more intensive livestock production systems in sub-Saharan Africa [7], notably in areas with high agricultural potential. Cattle breeding has an important place in the economy and socio-cultural activities of populations. Thus, controlling trypanosomoses and their vectors becomes a major step to increase the productivity of livestock production systems and contribute to small producers' food security. Several methods are proposed for the control and the reduction of trypanosomosis negative impacts on animal production. However, the impact of the present instability of tsetse flies natural habitats caused by climatic changes and anthropic pressure must be taken into consideration [6]. The global objective of this survey was to analyze the relative impact of production practices, particularly transhumance, and climatic conditions on the epidemiology of AAT and their main vectors (tsetse) in Burkina Faso.

## Materials and Methods

### 1. Study area

**Figure 1:**
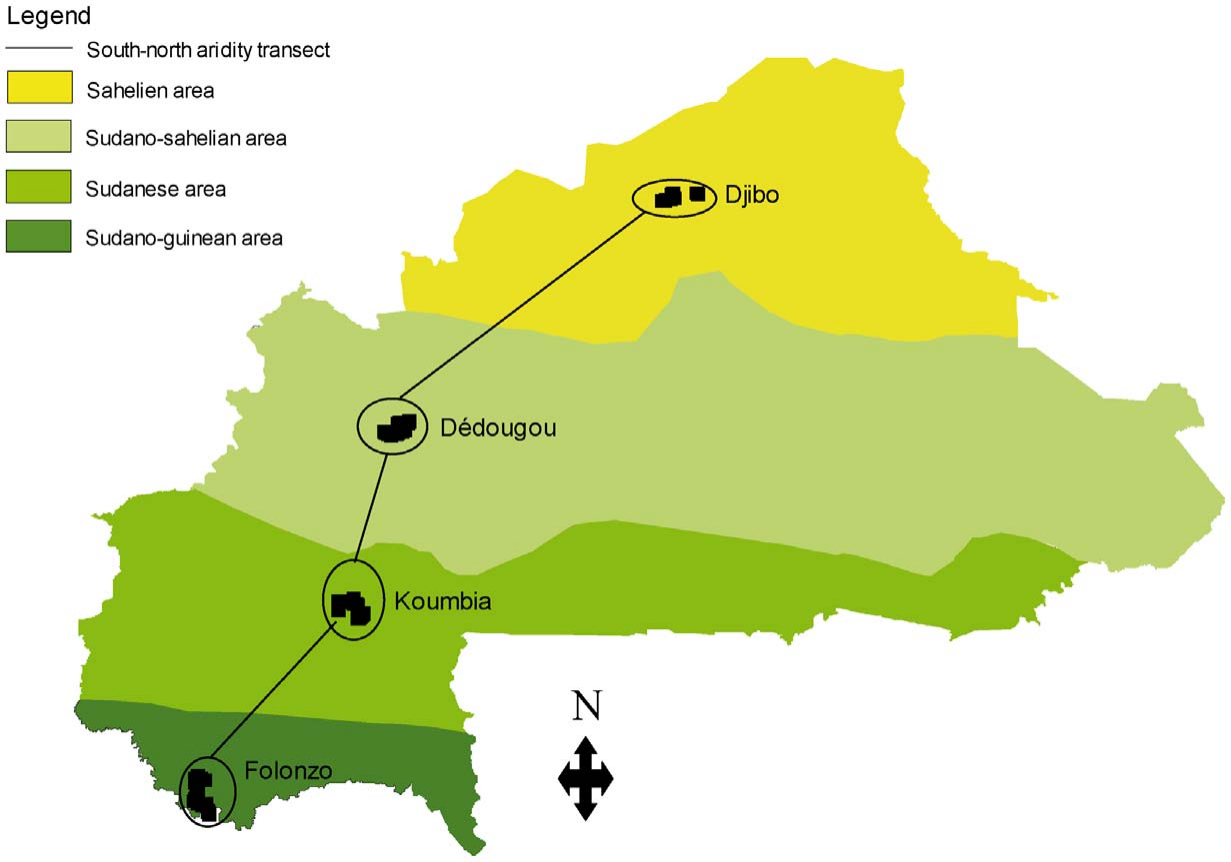
Location of the study sites along the climatic gradient in Burkina Faso.

The survey was achieved in four sites along a South-North transect in Burkina Faso (Fig. 1). These sites were selected according to cattle production systems and the agro-ecological area to which they belong: Folonzo, in the Sudano-Guinean area (16°60′W and 9°87′N), which is a welcome area for transhumant herds; Koumbia, in the Sudanese area (15°50′W and 11°07′N), which is a welcome, transit and attachment area for transhumant herds; Dédougou, in the Sudano-Sahelian area (15°55′W and 12°49′N), a welcome, transit and attachment area for transhumant herds; Djibo, in the Sahelian area (13°60′W and 14°10′N), an attachment area for transhumant herds.

### 2. Parasitological surveys

Blood samples were taken at the jugular vein from a total of 1,041 cattle (Table 1). In each livestock rearing system, cattle were selected randomly without criteria of age, sex or breed. However, the sex and the age of the animals, the date of the last treatment against AAT and the type of trypanocid used were recorded. The sample was composed of Fulani zebus and half-bred between Fulani zebus and “Baoulé” trypanotolerant cattle and in majority by females (73.63%). Animals belonged mainly to Fulani cattle farmers.

**Table 1:** Distribution of sampled animals in sites according to cattle rearing systems.

	Sites				
Cattle rearing system	Djibo	Dédougou	Koumbia	Folonzo	Total
Long transhumance	59	55	164	-	**278**
Short transhumance	178	-	-	63	**241**
Sedentary	159	130	200	33	**522**
**Total**	**396**	**185**	**364**	**96**	**1041**

Sites are ordered by increasing annual rainfall from the left to the right.

- Non identified cattle rearing system.

Two diagnosis methods were used for blood examination of the samples: the *Buffy-coat* (BCM) [8] to detect active infections by trypanosomes and the indirect ELISA- method to detect anti-trypanosomes antibodies in plasma, attesting past or present infections of the animal with trypanosomes [9]. The BCM allowed the diagnosis of trypanosome species based on morphological, mobility and size criteria [8].

### 3. Entomological surveys

All biting flies were trapped, immediately followed by tsetse flies dissection. In each of the 4 sites (Fig. 1), ten standard biconical traps [10] were set at intervals of 100 meters along cattle watering points. The trapping lasted 72 hours with harvests every 24 hours. Tsetse and other biting flies were numbered by species, by trap, and ADT (apparent density per trap per day) was calculated.

Non teneral tsetse flies were dissected using a binocular microscope. The dissection started with the proboscis, then the salivary glands and finally the mid-gut. After dissection, these organs were placed between a slide and a cover slip in a drop of Ringer's solution, and then observed using a microscope (×40) for parasite detection. Flies with an infected organ were collected in eppendorf tubes of 0.5 ml containing 30 µl of sterile distilled water (each organ independently) and stored at −20°C for ulterior PCR (Polymerase Chain Reaction) analysis, to determine the species of trypanosome involved, with primers for *T. vivax* and *T. congolense* savannah type [11]. In the same way, females' physiological age was determined by the dissection of the reproductive system [12].

### 4. Statistical analyses

Rates of infection of cattle and tsetse in different sites were compared using the Khi square test and binomial mixed effects models, where the cattle rearing system and the site represented the stationary effects. The herd was considered as a random effect.

The ADP were compared overall using a Kruskal-Wallis rank sum test [13] and then by pairs using the Steel-type none parametric multiple comparisons test (npmc) [14]. Tests were performed using the R-2.9.2-win32 software.

The mean number of infectious flies by trap and by day was calculated as an indicator of cyclical transmission risk [15]. The relative risks and their confidence intervals were obtained from bootstrapping in the ADT and infectious rate distributions from each site, assuming spatial homogeneity within a given site (5 000 Monte Carlo simulations, @risk software).

## Results

### 1. Parasitological surveys

Parasitological and serological prevalences were different between sites (p<0.05). They gradually increased along the climatic gradient of the Sahelian area toward the Sudano-Guinean area (Table 2), from 0 to 20% and 18 to 83% for parasitological and serological prevalence respectively (p<10^−3^).

**Table 2:** Parasitological and serological prevalences by trypanosome species according to cattle rearing systems and sites.

	Parasitological prevalences (%)									Serological prevalences (%)								
Site	Djibo			Dédougou**		Koumbia**		Folonzo*		Djibo			Dédougou**		Koumbia**		Folonzo*	
*CRS*	*Lt*	*St*	*sed*	*Lt*	Sed	*Lt*	*Sed*	*St*	*Sed*	*Lt*	*St*	*Sed*	*Lt*	*Sed*	*Lt*	*Sed*	*St*	*Sed*
*Tv*	0	0	0	0	0.8	0.6	10.5	9.5	3.0	3.4	9.0	14.5	12.7	22.3	40.8	27.5	28.6	63.6
*Tc*	0	0	0	0	0	0.6	0	12.7	18.2	5.1	6.2	8.8	25.5	15.4	19.5	31	50.8	27.2
*Tbb*	0	0	0	0	0	0	0	0	0	6.8	0.6	0.6	0	5.4	2.4	7.5	0	0
***T.spp/CRS***	**0**	**0**	**0**	**0**	**0.8**	**1.2**	**10.5**	**22.2**	**21.2**	**13.6**	**15.2**	**23.3**	**34.5**	**42.3**	**62.2**	**64.5**	**79.7**	**90.9**
***T.spp/Site***	**0**			**0.54**		**6.32**		**19.79**		**18.43**			**40.02**		**63.46**		**83.33**	

Sites are ordered by increasing annual rainfall from the left to the right.

**CRS:** Cattle rearing system; **St:** Short transhumance; **Lt:** Long transhumance; **Sed**: sedentary;

* long transhumance not identified in this site;

** short transhumance not identified in this site;

***Tv***: *Trypanosoma vivax*; ***Tc*** *: T. congolence*; ***Tbb*** *: T. brucei brucei*; ***T.spp***: all trypanosome species together.

Parasitological investigations allowed detecting active infections by *Trypanosoma vivax* and *T. congolense* with predominance of *T. vivax* in Dédougou and Koumbia and of *T. congolense* in Folonzo. No case of active infection by *T. brucei brucei* was identified in any of the sites (Table 2). In Djibo, no case of active infection by any trypanosome species was found.

The cattle rearing system had a significant impact on the parasitological prevalence (p = 0.02) but not on the serological prevalence (p = 0.77).

In Dédougou and Koumbia, parasitological prevalences were significantly higher in the sedentary rearing system that in those practicing long transhumance toward the subhumid and humid areas of the south (p<0.05). In Djibo, animals carriers of anti-trypanosomes antibodies were identified in the three cattle rearing systems (Table 2). *T. congolense* was predominant in the transhuming herds in all the sites (p<10^−3^).

In the whole area, the mean hematocrit of seropositive animals was not significantly different (p>0.05) from that of seronegatives: 32.63% and 33.04% in Djibo respectively, 30.63% and 31.35% in Dédougou, 32.58% and 32.92% in Koumbia and 25.77% and 28.13% in Folonzo.

### 2. Entomological surveys

**Figure 2:**
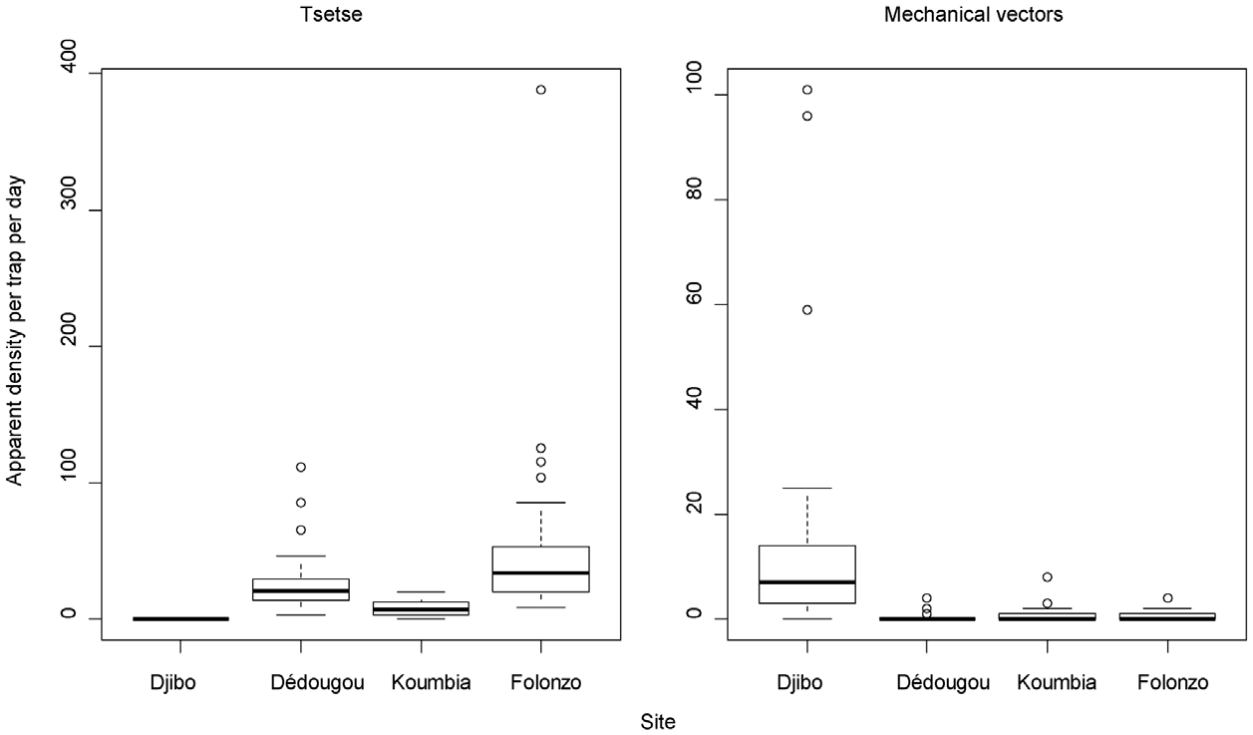
Distribution of biting flies along the climatic gradient in Burkina Faso. Sites are ordered by increasing annual rainfall from the left to the right. Boxplots present the median (bold line), quartiles (boxes), 95% confidence intervals (horizontal lines) and erratic values (dots).

Tsetse flies densities varied significantly along the climatic gradient (p<10^−3^). No tsetse was captured in Djibo, contrary to Folonzo where the ADT was 53.10±70.15; 8.16±5.55 in Koumbia and 27.24±23.72 in Dédougou (Fig. 2). The difference between Koumbia and Dédougou was not significant (p = 0.09) whereas all the other pair-comparisons were highly significant (p<0.02).

The specific density analysis shows that *Glossina tachinoides* has the largest spectrum of distribution. The highest ADT for this species, 39.63±56.69, found in Folonzo, was significantly higher than in Dédougou (21.62±20.18, p<0.05) and Koumbia sites (7.03±5.34, p<0.001). The ADT of *G. tachinoides* in Dédougou was also significantly higher than in Koumbia (p<0.001). *G. palpalis gambiensis* was present in 3 sites, but with lower ADT (1.87±2.71 in Koumbia, 0.36±0.76 in Dédougou and 0.79±1.28 in Folonzo) comparing to those of *G. tachinoides*. *G. morsitans submorsitans* and *G. medicorum* were found only in Folonzo with ADT of 10.5±15.23 and 0.6±1.30 respectively. The tsetse flies diversity decreased with the aridity gradient, from 4 to 0. At the opposite, the diversity of other biting flies increased from 1 to 4 with the aridity gradient (Table 3), and the apparent densities were higher (p<0.001) in the drier site (Djibo) than in the three other sites, where they were similar (p>0.05).

**Table 3:** Apparent densities of mechanical vectors along the climatic gradient.

Site	Djibo	Dédougou	Koumbia	Folonzo
*Atylotus agrestis*	14.81(23.95)	0(0)	0(0)	0.53(0.90)
*Tabanus sufis*	0.23(0.51)	0.28(0.84)	0(0)	0(0)
*Tabanus taeniola*	0.35(0.85)	0(0)	0.07(0.26)	0(0)
*Tabanus gratus*	0(0)	0(0)	0.66(1.56)	0(0)
*Chrysops distinctipennis*	0(0)	0.03(0.19)	0(0)	0(0)
*Stomoxys niger*	1.08(4.33)	0(0)	0.21(0.62)	0(0)
ADT (all species)	16.46(26.87)	0.31(0.85)	0.93(1.62)	0.53(0.90)
Species richness	4	2	3	1
Trap*days	26	29	29	30

Sites are ordered by increasing annual rainfall from the left to the right.

**Figure 3:**
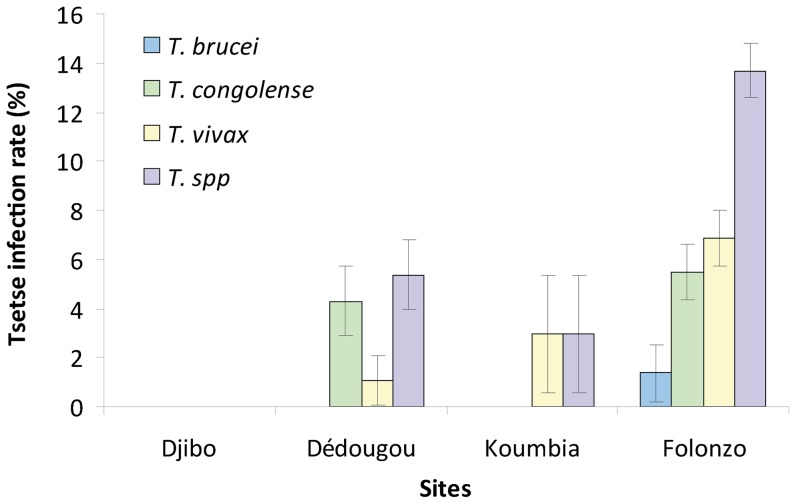
Tsetse infection rates along the aridity gradient. Sites are ordered by increasing annual rainfall from the left to the right.

The tsetse infection rates were significantly different between Dédougou (93 flies dissected), Koumbia (34) and Folonzo (146) (p = 0.038). Rates of infections were 9.5% (s.d. 1.8%) by microscopy and 4.8% (s.d. 1.3%) only by PCR (p<0.05), suggesting that almost half of the flies were infected by non pathogenic species of trypanosomes for cattle. The highest proportion of flies infected, all trypanosomes species together, was observed in Folonzo (13.70%; p = 0.03), by comparison to those in Koumbia (2.94%) and Dédougou (5.38%) (Fig. 3). Tsetse infections by *T. brucei brucei* were observed exclusively in Folonzo in *G. tachinoides* (1.37%).

Overall, the risk of cyclical transmission, measured as the apparent density of infectious fly per trap per day, was 1.18 [95%CI 0–3.65] in Dedougou, 0.31 [0.07–0.67] in Koumbia and 7.27 [3.66–12.59] in Folonzo. It was thus 23.04 [2.80–122.29] times more important in Folonzo than it the two other sites (p<0.05), between which it was not significantly different (p>0.05).

The physiological age of 59 tsetse in Dédougou, 23 in Koumbia and 43 in Folonzo were measured. The mean age was significantly lower in the site of Koumbia (28±18 days) than the two others (p = 0.007). There was no difference between the mean age of the flies in Dédougou (39±20 days) and Folonzo (38±13 days) (p = 0.74).

## Discussion

The importance of AAT prevalence decreased with the aridity degree in our study area, with the exception of Dédougou where the Mouhoun river loop allows the persistency of high tsetse densities [15], [16]. The fact that some sites are more infected than others demonstrates the spatial heterogeneity of the AAT along of climatic gradient.

The predominance of *T. vivax* over *T. congolense* in Koumbia and in Dédougou could be related to a better transmission by riverine tsetse [17]. On the contrary, the high prevalence of *T. congolense* in Folonzo reveals the importance of contacts between animals and *G. morsitans submorsitans*, known as efficient vectors for this trypanosome [18], [19]. This hypothesis is reinforced by the fact that, at the time of the entomological survey, *G. morsitans submorsitans* was found abundant in the area (10.5±15.23 tsetse/trap/day). The absence of *T. brucei brucei* in cattle and its weak seroprevalence, confirms tendencies observed in recent parasitological studies conducted in Burkina [20], [21]. In the study of Dayo et al. (2009), *T. congolense* was also found much predominant in cattle, in a site close to Folonzo.

Three typical cycles have been described to characterize the epidemiological settings of African Animal Trypanosomosis, including sylvatic trypanosomosis, interface trypanosomoses and endemic livestock trypanosomosis. Koumbia and Dédougou can be considered as within the endemic livestock trypanosomosis cycle, where cattle are the main hosts and wild fauna tends to disappear. On the contrary, Folonzo represents a typical interface trypanosomoses situation where the wild fauna is still abundant and the pressure of cyclical vectors very high. It would be interesting to investigate whether the strains of *T. vivax* transmitted in the letter cycle are more virulent than in the two other sites, as observed for *T. congolense* in Southern Africa [22]. The higher parasitological prevalence in sedentary animals in Dédougou and Koumbia could be explained by higher trypanocid treatment frequencies in transhumant cattle. Even if this was not evidenced from interviews with livestock owners, previous studies have found that such information can be very unreliable and often many more treatments can be given than would be apparent. The resident cattle are concentrated along the last water points (Mouhoun and Bougouriba respectively), and experience high contact intensity with riverine tsetse that are good vectors of *T. vivax*. This transmission might be seasonally relayed by mechanical vectors, particularly abundant in late rainy season and cold dry season in our study area (September-November) [23]. Similar observations were done in Gambia using parasitological follow-up of transhumant and sedentary animals [24].

The absence of parasitological prevalence in all cattle rearing systems in Djibo might be explained by the combined effect of the absence of cyclical vectors and the frequency of trypanocid treatments by the breeders (before, during and after the return from transhumance in the trypanosomian endemic area). This site is located far upon the northern limit of tsetse, which was very well characterized during former surveys in the study area [4], [16], [25]. The majority of antibodies detected in animals in Djibo probably result from past infections. This hypothesis is supported by the absence of significant difference between the hematocrit of seropositive and seronegative animals. Sedentary and low transhumance herds are probably contaminated locally by the mechanical vectors from animals infected during their transhumance in the endemic AAT area. Indeed, the letter could maintain an intermittent epidemic transmission of AAT [26], explaining the serological predominance of *T. vivax* in this site. All these animals (sedentary animals infected mechanically and animals infected during transhumance) are treated several times during the year and were exempt of parasites during the survey, but remain seropositive for a long time (∼3 months) [9]. This migratory situation, that always existed, could be exemplified by the climatic accidents and anthropic pressure effects that increase herd movements [6]. The sedentary herds in Djibo cannot be considered as within the three epidemiological cycles formerly described [6] and we thus hereby make the hypothesis of a fourth cycle named “epidemic mechanical livestock trypanosomosis” where local transmission is seasonal and ensured by mechanical vectors, where cattle are the main hosts, and where trypanosomes are brought in by cattle coming back from transhumance in the tsetse infested area.

With a mean apparent density of infectious tsetse of 0.74 [95% CI 0.04–2.01], the cyclical transmission risk can be considered as high in Dédougou and Koumbia, according to former studies [15], , and very high in Folonzo 7.27 [3.66–12.59]. This observation, as well as the growing diversity of tsetse species from the Sahelian to the Sudano-Guinean area is probably related to climatic conditions (rainfall and temperature) [6], the fragmentation degree of vegetal formations caused by the demographic pressure [4] and the presence of wild fauna in Folonzo.

Nowadays, tsetse flies have regressed in the sahelian area where they have been captured until 1935 [25]. The presence of permanent hydrographic network, as the Mouhoun river loop in Dédougou [15], or the Niayes in Senegal, however allow riverine tsetse to persist in high densities in some sahelian sites [16], [28], as observed in this survey in Dédougou. Tsetse flies absence in Djibo is attributable to the modification of the hygrometric conditions and temperatures (drought episodes of 1970–1990) associated to anthropic pressure on the plant and animal resources leading to the disappearance of tsetse forest habitats and wild hosts. The same factors lead to the disappearance of *G. morsitans submorsitans* in Dédougou and Koumbia [29]. Riverine flies, however, seem more resilient thanks to an opportunistic feeding behaviour associated to learning capacities [30] and linear dispersal along rivers [31].

The reduction of the mean age of the tsetse population in Koumbia indicates a higher adult mortality, which is probably a result of the perturbation/deterioration of their habitat. The reduction of the lifespan also favors trypanosome species having a short extrinsic cycle life as *T. vivax* (10 days), in comparison to those with longer cycle as *T. congolense* (14 days) and *T. brucei brucei* (30 days) [32].

Thus, this study confirmed that aridity and landscape fragmentation are associated to a reduction of the biodiversity of hosts, cyclical vectors and transmitted parasites in Burkina Faso [6]. Moreover, the impact of tsetse appeared not limited to their distribution area, but extended to a buffer area corresponding to the transhumance radius of herds, where transmission is probably relayed by mechanical vectors. The situation documented here only partially contributes to characterizing the impact of production systems and climate on tsetse populations and prevalence of AAT in Burkina Faso. It would be interesting to investigate whether similar situations are encountered elsewhere and using other methods (namely monitoring of AAT incidence).

## References

[1] Müller C (2009) Climate change impact: on Sub-Saharan Africa an overview and analysis of scenarios and models. Bonn: Deutsches Institut für Entwicklungspolitik. 47 p.

[2] Hulme M, Doherty R, Ngara T, New M, Lister D (2001) African climate change: 1900–2100. Clim Res 17: 145–168.

[3] Courtin F, Guengant JP (2011) Un siècle de peuplement en Afrique de l'Ouest. Nat Sci Soc 19: 256–265.

[4] Guerrini L, Bord JP, Ducheyne E, Bouyer J (2008) Fragmentation analysis for prediction of suitable habitat for vectors: the example of riverine tsetse flies in Burkina faso. J Med Entomol 45: 1180–1186. 19058646 10.1603/0022-2585(2008)45[1180:fafpos]2.0.co;2

[5] TerraAfrica (2009) Land climate: the role of sustainable land management for climate change adaptation and mitigation in Sub-Saharan Africa. Namibie: NEPAD Planning and Coordinating Agency. 110 p.

[6] Van den Bossche P, de La Rocque S, Hendrickx G, Bouyer J (2010) A changing environment and the epidemiology of tsetse-transmitted livestock trypanosomiasis. Trends Parasitol 26(5): 236–243. 20304707 10.1016/j.pt.2010.02.010

[7] Swallow BM (1999) Impacts of Trypanosomiasis on African Agriculture. Rome: FAO. 46 p.

[8] Murray M, Murray PK, McIntyre WI (1977) An improved parasitological technique for the diagnosis of African trypanosomiasis. Trans R Soc Trop Med Hyg 71: 325–326. 563634 10.1016/0035-9203(77)90110-9

[9] Desquesnes M (1997) Evaluation of a simple PCR technique for the diagnosis of *trypanosoma vivax* infection in the serum of cattle, in comparison to parasitological techniques and antigen-enzyme-linked immuno sorbent assay. Acta Trop 65: 139–148. 9177575 10.1016/s0001-706x(96)00643-2

[10] Challier A, Laveissière C (1973) Un nouveau piège pour la capture des glossines (*Glossina:* Diptera, Muscidae): description et essais sur le terrain. Cah ORSTOM, sér Ent Méd et Parasitol 10: 251–262.

[11] Desquesnes M, Davila AMR (2002) Applications of PCR-based tools for detection and identification of animal trypanosomes: a review and perspectives. Vet Parasitol 109: 213–231. 12423934 10.1016/s0304-4017(02)00270-4

[12] Itard J (1966) Cycle de l'oogenèse chez les femelles de Glossina tachinoides Westw. et détermination de l'âge physiologique. Rev Elev Méd vét Pays trop 19: 331–350. 5929419

[13] Hollander M, Wolfe DA (1973) Non parametric statistical inference. New York: John Wiley & Sons. 115–120 p.

[14] Munzel U, Hothorn LA (2001) A Unified Approach to Simultaneous Rank Test Procedures in the Unbalanced One-way Layout. Biom J 43: 553–569.

[15] Bouyer J, Guerrini L, Desquesnes M, de la Rocque S, Cuisance D (2006) Mapping African Animal Trypanosomosis risk from the sky. Vet Res 37: 633–645. 16777035 10.1051/vetres:2006025

[16] Bouyer J, Guerrini L, César J, de la Rocque S, Cuisance D (2005) A phyto-sociological analysis of the distribution of riverine tsetse flies in Burkina Faso. Med Vet Entomol 19: 372–378. 16336302 10.1111/j.1365-2915.2005.00584.x

[17] Reifenberg JM, Cuisance D, Frezil JL, Cuny G, Duvallet G (1997) Comparison of the susceptibility of different Glossina species to simple and mixed infections with Trypanosoma (Nannomonas) congolense savannah and riverine forest types. Med Vet Entomol 11: 246–252. 9330255 10.1111/j.1365-2915.1997.tb00402.x

[18] Merid N, Melaku G, Emiru S (2007) Epizootiological importance of Glossina morsitans submorsitans (Diptera: Glossinidae) (Newstead) in the Ghibe River Valley, Southwest Ethiopia. Acta Trop 102: 100–105. 17543265 10.1016/j.actatropica.2007.04.004

[19] Cherenet T, Sani RA, Panandam JM, Nadzr S, Speybroeck N, et al (2004) Comparison of the susceptibility of different Glossina species to simple and mixed infections with Trypanosoma (Nannomonas) congolense savannah and riverine forest types. Med Vet Entomol 11: 246–252. 10.1111/j.1365-2915.1997.tb00402.x 9330255

[20] Dayo GK, Bengaly Z, Messad S, Bucheton B, Sidibe I, et al (2010) Prevalence and incidence of bovine trypanosomosis in an agro-pastoral area of southwestern Burkina Faso. Res Vet Sci 88: 470–477. 20044115 10.1016/j.rvsc.2009.10.010

[21] Bouyer J, Stachurski F, Gouro A, Lancelot R (2009) Control of bovine trypanosomosis by restricted application of insecticides to cattle using footbaths. Vet Parasitol 161: 187–193. 19231084 10.1016/j.vetpar.2009.01.018

[22] Van Den Bossche P, Chitanga S, Masumu J, Marcotty T, Delespaux V (2011) Virulence in Trypanosoma congolense Savannah subgroup. A comparison between strains and transmission cycles. Parasite Immunol 33: 456–460. 21204855 10.1111/j.1365-3024.2010.01277.x

[23] Koné N, N'Goran EK, Sidibé I, Kombassere AW, Bouyer J (2011) Spatio-temporal distribution of tsetse (Diptera: Glossinidae) and other biting flies (Diptera: Tabanidae and Stomoxinae) in the Mouhoun River Basin, Burkina Faso. Med Vet Entomol 25: 156–168. 21198714 10.1111/j.1365-2915.2010.00938.x

[24] Wacher TJ, Rawlings P, Snow WF (1993) Cattle migration and stocking densities in relation to tsetse-trypanosomosis challenge in the Gambia. Trop Med Parasitol 87: 517–524. 10.1080/00034983.1993.11812804 8311578

[25] Courtin F, Rayaissé J-B, Tamboura I, Serdébéogo O, Koudougou Z, et al (2010) Updating the Northern Tsetse Limit in Burkina Faso (1949–2009): Impact of Global Change. Int J Environ Res Public Health 7: 1708–1719. 20617055 10.3390/ijerph7041708 PMC2872350

[26] Desquesnes M, Biteau-Coroller F, Bouyer J, Dia ML, Foil LD (2009) Development of a mathematical model for mechanical transmission of trypanosomes and other pathogens of cattle transmitted by tabanids. Int J Parasitol 39: 333–346. 18755195 10.1016/j.ijpara.2008.07.004

[27] Guerrini L, Bouyer J (2007) Mapping African Animal Trypanosomosis risk: the landscape approach. Vet Ital 43: 643–654. 20422544

[28] Bouyer J, Seck MT, Sall B, Guerrini L, Vreysen MJB (2010) Stratified entomological sampling in preparation of an area-wide integrated pest management programme: the example of *Glossina palpalis gambiensis* in the Niayes of Senegal. J Med Entomol 47(4): 543–552. 20695269 10.1093/jmedent/47.4.543 PMC7027262

[29] Rouamba J, Jamonneau V, Sidibé I, Solano P, Courtin F (2009) Impact de la dynamique du peuplement sur la distribution des glossines dans la boucle du Mouhoun (Burkina Faso). Parasite 16: 11–19. 19353947 10.1051/parasite/2009161011

[30] Bouyer J, Pruvot M, Bengaly Z, Guerin PM, Lancelot R (2007) Learning influences host choice in tsetse. Biol Let 3: 113–116. 17251119 10.1098/rsbl.2006.0578 PMC2375919

[31] Bouyer J, Balenghien T, Ravel S, Vial L, Sidibé I, et al (2009) Population sizes and dispersal pattern of tsetse flies: rolling on the river? Mol Ecol 18: 2787–2797. 19457176 10.1111/j.1365-294X.2009.04233.x

[32] Cuisance D, Itard J, Desquesnes M, Frézil JL, de La Rocque S (2003) Trypanosomoses, Epidémiologie. In: Tec et Doc, Editions Médicales Internationales, editors. Principales maladies infectieuses et parasitaires du bétail Europe et Régions chaudes. Londres - Paris - New York: Lavoisier. pp. 1627–1650.

